# An Introductory Lecture, Delivered at the Opening of the Thirty-First Session of the Medical College of Ohio

**Published:** 1851-04

**Authors:** 


*An Introductory Lecture, delivered at the Opening of the Thirty-first Session of the Medical College of Ohio, Nov.* 4, 1850. By John Bell, M. D., Professor of the Theory and Practice of Medicine, &c.

The author has sustained his well earned reputation as a clear thinker and forcible writer. Such lectures as this of Dr. Bell must produce, ultimately, a good effect upon the students and promiscuous audiences who hear them. We have space only for a paragraph, but it is a good one. Oh! that it may find favor where it ought to have effect; but the age of selfishness has not yet yielded to the millennium of self-denial.

"It should be the aim of the different [several ?] medical schools of the country, in a spirit of generous rivalry, to raise the standard, while increasing the means, of medical education. They must carefully reject all the expedients to swell the number of their classes, which smack of the petty and not always conscientious trafficker, rather than the elevation of true science, and the sensitiveness of refined literature. Encouragements held out to the illiterate, the unstable, and it may be the more unworthy still, to study medicine, are premiums given to ignorance and indolence. Many a good mechanic and thrifty farmer are thus lost, in making them bad doctors."

There is the unexceptionable precept, who will show the first example? We will wait ten years for an answer.

